# The early phase transcriptome of bovine monocyte-derived macrophages infected with *Staphylococcus aureus in vitro*

**DOI:** 10.1186/1471-2164-14-891

**Published:** 2013-12-17

**Authors:** Anna Monika Lewandowska-Sabat, Guro Margrethe Boman, Alison Downing, Richard Talbot, Anne Kristine Storset, Ingrid Olsaker

**Affiliations:** 1 Department of Basic Sciences and Aquatic Medicine, Norwegian School of Veterinary Science, P.O. Box 8146 Dep, NO-0033 Oslo, Norway; 2 ARK-Genomics, The Roslin Institute and Royal (Dick) School of Veterinary Studies, University of Edinburgh, Roslin, Midlothian EH25 9PS, UK; 3 Department of Food Safety and Infection Biology, Norwegian School of Veterinary Science, NO-0033 Oslo, Norway

**Keywords:** Alternative activation, Cattle, Intracellular, Macrophages, Mastitis, Microarray, *Staphylococcus aureus*

## Abstract

**Background:**

In the mammary gland, local recruitment and action of macrophages is a key immunological defence mechanism against infection. Macrophages are members of the innate immune system, serve as the first line of the defence against invading pathogens and are critical effectors and regulators of inflammation. We have examined the early phase response of bovine macrophages to infection with live *Staphylococcus aureus*. Genome-wide transcript profiling of blood monocyte-derived macrophages from six Norwegian Red heifers infected with live *S. aureus* for 2 and 6 hours *in vitro* was performed.

**Results:**

About 420 of the 17 000 genes on the ARK-Genomics bovine cDNA array were differentially regulated at 6 hours post infection. Approximately 70% of the responding genes had a known identity (Entrez Gene ID) and were used in the identification of overrepresented pathways and biological functions in the dataset.

Analysis of a subset of differentially regulated genes (List eQG) obtained by comparison with data from genome-wide association mapping in Norwegian Red cattle identified anti-inflammatory cytokines *interleukin 4* and *interleukin 13* as putative expression quantitative trait loci, suggesting that *S. aureus* infection triggers alternative activation of macrophages. Moreover, several classical activation pathways were found, mainly cellular immune response and cytokine signaling pathways, i.e. triggering receptor expressed on myeloid cells 1 (TREM1) and nucleotide-binding and oligomerization domain-like receptor (NLR) pathways. Tumor necrosis factor receptor superfamily member 5 (CD40 ligand) was identified as an upstream regulator which points toward CD40 likely acting as a co-stimulatory receptor during Toll-like receptor 2(TLR2)-mediated inflammatory response of bovine macrophages to *S. aureus* infection. Furthermore, peptidoglycan was identified as an upstream regulator in the List eQG, which indicates that this bacterial cell-wall component might be pivotal in macrophage intracellular bacterial recognition during early inflammation.

**Conclusions:**

Here we have shown that *in vitro* infection of bovine macrophages with live *S. aureus* induced both alternative and classical activation pathways. Alternative activation of macrophages may be a mechanism contributing to intracellular persistence of *S. aureus* in the course of inflammation such as during mastitis in dairy cattle.

## Background

Mastitis is an inflammatory condition affecting the mammary gland and the most frequent disease resulting in serious economic losses and herd management problems in dairy production. Bovine mastitis is caused by infection with pathogenic bacteria such as *Staphylococcus aureus*, *Streptococcus uberis* and *Escherichia coli*[1].

*S. aureus* is a widespread pathogen of relevance to both human and veterinary medicine. It is a major causative factor of bovine mastitis in Norway (and worldwide), causes clinical and subclinical intramammary infections and produces 200–300 virulence factors. It has been demonstrated that *S. aureus* invades and survives within mammalian host cells and is competent to replicate in the phagosome or escape the phagosome and persist within the host cells, which can induce anti-apoptotic programs in phagocytes [2]. Intracellular survival of *S. aureus* has been implied as an immune-evasive strategy causing chronic mastitis infections in cattle. Chronically infected animals are sources of recurrent infections and contribute to spreading *S. aureus* to other cows and herds.

Monocytes and macrophages are critical effectors and regulators of inflammation serving as the first line of innate defence against invading pathogens. During inflammation, circulating monocytes migrate from the blood to tissues in response to chemokine signaling where they differentiate into macrophages. Macrophages are capable of phagocytosis and production of both proinflammatory and anti-inflammatory cytokines [3]. In the bovine udder, macrophages are present in the mammary gland interstitium and alveolar cells, defending epithelium from invading pathogens [4]. Local recruitment and action of macrophages in the mammary gland is an essential immunological defence mechanism against infection.

In addition to the classical macrophage activation (M1) induced by interferon gamma (IFNg), where T helper 1 cell-type activation of macrophages triggers a pro-inflammatory response required to kill intracellular pathogens, macrophages also undergo alternative activation. Alternatively activated macrophages (M2) are characterized by suppressed production of proinflammatory cytokines, anti-inflammatory effects and reduced killing capacity toward pathogens [5,6]. Recently, M2 macrophages have been further divided into three subsets: M2a, induced by the T helper 2 cytokines interleukin 4 (IL-4) and IL-13 and referred to as wound-healing and tissue repair macrophages; M2b, a less understood group; and M2c, stimulated by IL-10 and referred to as regulatory macrophages and deactivators of the immune response [6,7]. It has been proposed that alternative activation of macrophages triggered by the intracellular pathogen *Francisella tularensis* is a mechanism by which bacteria can evade the host immune response to favor its intracellular survival [8].

Macrophages can also be activated through expression of macrophage surface receptor tumor necrosis factor superfamily member 5 (CD40) and its functional ligation with CD40 ligand (CD154) expressed on activated T helper cells. CD40 signaling in macrophages induces the nuclear factor kappa B (NFKB)-mediated synthesis of pro-inflammatory cytokines including IL-1a, IL-1b and tumor necrosis factor alpha (TNFa), and several chemokines [9]. It has been demonstrated that IFNB1 and TNFa proteins increase expression of CD40 protein in human endothelial cells and blood-derived dendritic cells [10,11], and NFKB complex increases expression of *CD40* in murine dendritic cells [12]. Moreover, studies of the intracellular pathogen *Toxoplasma gondii* have shown that CD40 signaling induces the TNFa-dependent antimicrobial activity of macrophages even in the absence of IFNg and production of reactive nitrogen intermediates, central elements of classically activated macrophages [13].

It has been suggested previously, that *S. aureus* cell wall peptidoglycan is a biological effector with different stimulatory activities for several pattern recognition receptors (PRRs) of immune cells such as Toll-like receptor 2 (TLR2) and cytosolic nucleotide-binding and oligomerization domain (NOD)-containing proteins. However, later studies have reported that *S. aureus* lipoproteins are ligands for TLR2 [14,15] and after engulfment of *S. aureus* by macrophages, the TLR2/TLR6 complex is recruited to phagosomes for an efficient lipoprotein and TLR2 interaction (reviewed in [16]). TLR2 uses a myeloid differentiation primary response gene 88 (MyD88)-dependent signaling pathway resulting in NFKB-mediated expression of pro-inflammatory cytokines and chemokines. However, binding of *S. aureus* peptidoglycan components to intracellular NOD2 protein leads to nuclear translocation of NFKB and induction of cytokine production and inflammation (reviewed in [17]).

The aim of this study was to evaluate an early phase gene response of bovine macrophages to infection with live *S. aureus.* Challenging macrophages with live bacteria induces several genes and pathways that are involved in immunological response likely also during intramammary infections. Transcript profiling of blood monocyte-derived macrophages challenged with *S. aureus* for 2 and 6 hours *in vitro* were assessed by microarray and the expression of selected genes was validated by reverse transcription-quantitative PCR (RT-qPCR).

## Results

### Microarray analysis

**Figure 1:**
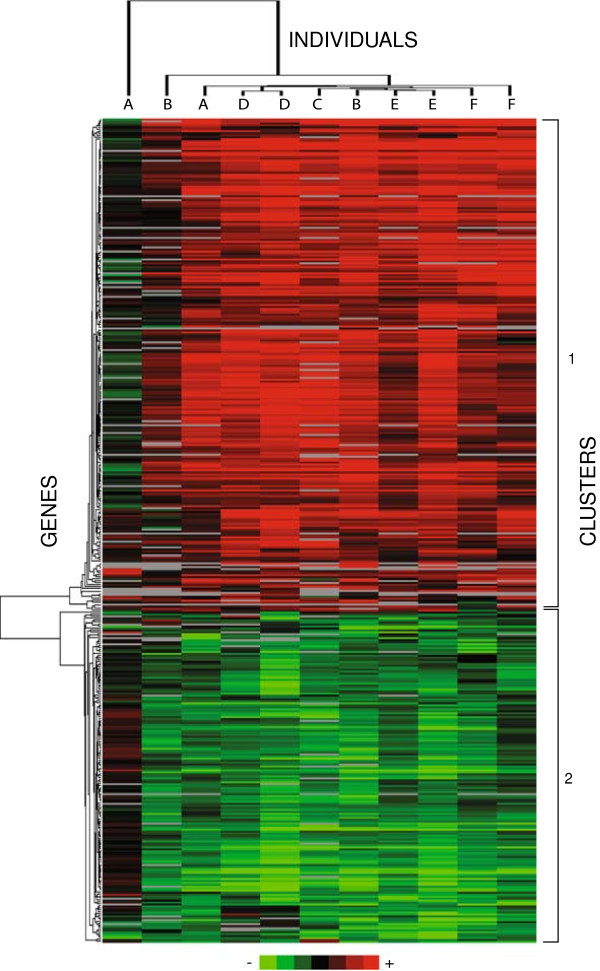
**Hierarchical clustering of 418 genes differentially expressed in macrophages in response to *Staphylococcus aureus *6 hours post infection.** Letters (A-F) denote the six Norwegian Red heifers tested in the study. Uninfected control samples of each individual were used as references, i.e. control and *Staphylococcus aureus* infected samples from one individual were hybridized to one array. Two dye-swaps (arrays) were used per individual except for individual C, where only one array was produced.

A total of 418 genes were found to be differentially expressed in the infected cells vs. uninfected control cells at 6 h post infection (~2.5% of the transcripts represented on the microarray). Of these, 250 genes were up-regulated, while 168 were down-regulated (Figure 1, Additional file 1: Table S1). Fold change values ranged from +11.2 to -4.3. Out of these 418 genes, 293 obtained gene IDs after Ensembl reannotation (~70%). Expression data are deposited in the Array Express Archive (accession: E-TABM-1133). Large variability in the responses between the individuals at the 2 h time point was observed resulting in no significant regulated genes at this time point (data not shown).

Most of the individuals showed a similar expression pattern at the 6 h time point. The dye-swap arrays clustered together for 3 individuals (D, E, F), but not for individuals A and B. Only one array (no dye-swap) was produced for individual C, which clustered with the remaining arrays (Figure 1). Two main clusters of genes were found and the first grouped the up-regulated genes and second grouped the down-regulated genes (Additional file 1: Table S1).

Database for Annotation, Visualization and Integrated Discovery (DAVID) functional annotation clustering revealed that 266 of the differentially expressed and Ensembl reannotated genes mapped to 14 different Kyoto Encyclopedia of Genes and Genomes (KEGG) pathways (*p* ≤ 0.05), mainly signaling in the immune response and apoptosis pathways. A subset of the differentially regulated genes (List eQG) with genomic positions linked to quantitative trait loci (QTL) affecting susceptibility to mastitis in Norwegian Red cattle (NRF) was identified previously [18]. Twenty-five of the List eQG genes were mapped to 5 different KEGG pathways (*p* ≤ 0.05), mainly signaling in immune response and cancer (Table 1). Ingenuity Pathway Analysis (IPA) mapped 281 gene IDs using the Ensembl reannotated differentially expressed genes on microarray, which resulted in identification of 170 canonical pathways (*p* ≤ 0.05, Additional file 2: Table S2a). When the List eQG was used, IPA mapped 25 gene IDs and 65 canonical pathways were identified (*p* ≤ 0.05, Additional file 2: Table S2b). The top canonical pathways for both gene lists are presented in Table 2.

**Table 1:** **Enriched Kyoto Encyclopedia of Genes and Genomes (KEGG) pathways generated by Database for Annotation, Visualization and Integrated Discovery (DAVID) (*p*** **≤ 0.05)**

	**KEGG pathway**	***p*-value**
**A**	NOD-like receptor signaling pathway*	4.9 × 10^-7^
	Cytokine-cytokine receptor interaction*	1.7 × 10^-6^
	Toll-like receptor signaling pathway*	6.2 × 10^-5^
	Asthma*	9.8 × 10^-4^
	T cell receptor signaling pathway	3.6 × 10^-3^
	Jak-STAT signaling pathway	4.1 × 10^-3^
	Apoptosis	4.4 × 10^-3^
	Chemokine signaling pathway	4.9 × 10^-3^
	Cytosolic DNA-sensing pathway	1.4 × 10^-2^
	Allograft rejection	2.6 × 10^-2^
	RIG-I-like receptor signaling pathway	3.0 × 10^-2^
	Sphingolipid metabolism	3.0 × 10^-2^
	Natural killer cell mediated cytotoxicity	4.8 × 10^-2^
	Fc epsilon RI signaling pathway	4.8 × 10^-2^
**B**	Cytokine-cytokine receptor interaction*	5.2 × 10^-4^
	Jak-STAT signaling pathway	2.8 × 10^-3^
	Colorectal cancer	1.4 × 10^-2^
	Toll-like receptor signaling pathway	1.8 × 10^-2^
	Pathways in cancer	2.6 × 10^-2^

A) The list of differentially regulated on microarray (n = 418) and Ensembl reannotated genes was used as an input for functional annotation clustering; B) Subset of these genes (n = 28; List eQG from [18]) was used as an input. Asterisk (*) denotes the significant KEGG pathways after the Benjamini–Hochberg multiple testing correction (*p* ≤ 0.05).

**Table 2:** Top canonical pathways generated by Ingenuity Pathway Analysis (IPA)

	**Ingenuity canonical pathways**	***p*-value**	**Ratio**	**Molecules**
**A**	TREM1 signaling	2.71 × 10^-11^	1.69 × 10^-1^	IL8, CXCL3, ICAM1, CD40, GRB2, IL10, TLR8, CASP1, IL1B, FCGR2B, NFKB1, TNF
	Hepatic fibrosis/hepatic stellate cell activation	8.32 × 10^-11^	1.10 × 10^-1^	CXCL3, IL8, IL4R, CCR5, ICAM1, CD40, MYL6, IL10, IL6R, IL1B, IFNGR1, CCL5, NFKB1, TNF, CCR7, IL4
	IL-10 signaling	3.82 × 10^-9^	1.41 × 10^-1^	TRAF6, SOCS3, FOS, IL4R, CCR5, MAPK14, IL10, IL1B, FCGR2B, NFKB1, TNF
	IL-12 signaling and production in macrophages	6.87 × 10^-9^	8.97 × 10^-2^	IL10, MAF, IFNGR1, NFKB1, IRF1, TRAF6, FOS, MAPK14, CD40, ZNF668, NOS2, TNF, PRKD3, IL4
	IL-17A signaling in gastric cells	3.33 × 10^-8^	2.80 × 10^-1^	IL8, FOS, MAPK14, CCL20, CCL5, NFKB1, TNF
**B**	Role of cytokines in mediating communication between immune cells	8.88 × 10^-7^	7.27 × 10^-2^	IL8, IFNB1, IL13, IL4
	Communication between innate and adaptive immune cells	6.73 × 10^-6^	3.67 × 10^-2^	IL8, IFNB1, CCR7, IL4
	Airway inflammation in asthma	1.81 × 10^-5^	3.33 × 10^-1^	IL13, IL4
	Remodeling of epithelial adherens junctions	1.08 × 10^-4^	4.41 × 10^-2^	ACTR3, MAPRE1, CLIP1
	Prolactin signaling	1.5 × 10^-4^	3.75 × 10^-2^	SOCS3, FOS, IRF1

A) The list of differentially regulated on microarray (n = 418) and Ensembl reannotated genes was used as an input for IPA; B) Subset of these genes (n = 28; List eQG from [18]) was used as an input.

Top molecular and cellular functions identified by IPA for differentially expressed genes on microarray and List eQG were similar and were associated with death, survival, function and movement of immune cells (Table 3). All biological functions identified in IPA for both datasets are presented in Additional file 3: Table S3.

**Table 3:** Top molecular and cellular functions identified by Ingenuity Pathway Analysis (IPA)

	**Top molecular and cellular functions**	***p*-value**	**No. of molecules**
**A**	Cell death and survival	5.86 × 10^-17^ – 8.33 × 10^-6^	114
	Cellular function and maintenance	5.21 × 10^-16^ – 5.86 × 10^-6^	73
	Cellular movement	1.79 × 10^-13^ – 7.87 × 10^-6^	68
	Cellular development	2.52 × 10^-13^ – 6.75 × 10^-6^	105
	Cellular growth and proliferation	2.52 × 10^-13^ – 8.04 × 10^-6^	111
**B**	Cell death and survival	7.73 × 10^-10^ – 9.61 × 10^-4^	14
	Cellular development	1.35 × 10^-9^ – 8.97 × 10^-4^	18
	Cellular growth and proliferation	1.35 × 10^-9^ – 8.97 × 10^-4^	18
	Cellular movement	1.82 × 10^-9^ – 9.32 × 10^-4^	14
	Cellular function and maintenance	1.06 × 10^-8^ – 9.32 × 10^-4^	15

A) The list of differentially regulated on microarray (n = 418) and Ensembl reannotated genes was used as an input for IPA; B) Subset of these genes (n = 28; List eQG from [18]) was used as an input.

**Figure 2:**
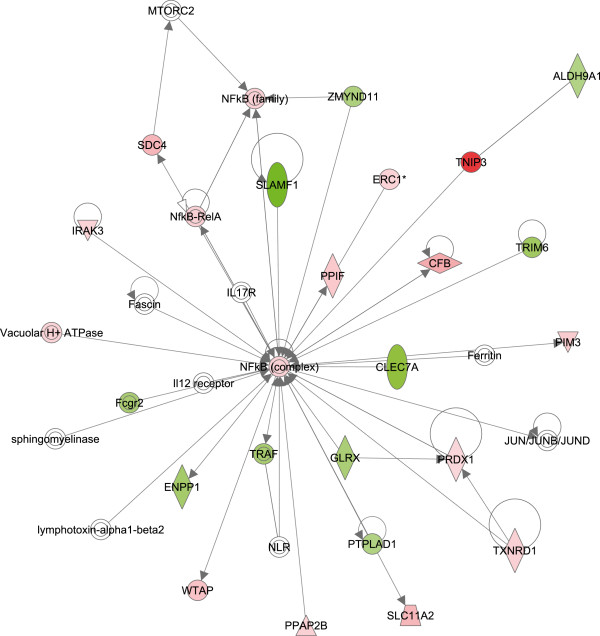
**Functional network over-represented in a list of differentially expressed genes on the microarray involving *nuclear factor kappa B *(*NFKB*) complex and associated molecules with a network score of 31, as identified by Ingenuity Pathway Analysis (IPA).** Red denotes molecules that were up-regulated and green denotes molecules down-regulated in response to 6 hours infection with live *Staphylococcus aureus* in bovine macrophages.

**Figure 3:**
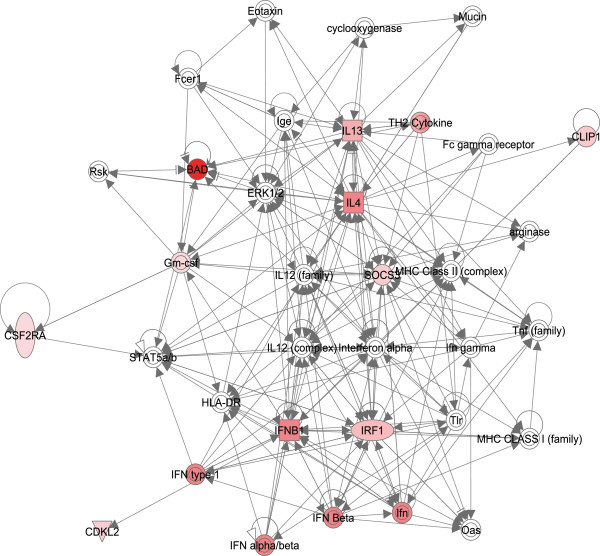
**Functional network over-represented in the subset of the differentially regulated genes (List eQG) involving *interleukin 4 *(*IL-4*), *IL-13 *and *suppressor of cytokine signalling 3 *(*SOCS3*) and associated molecules with a network score of 20, as identified by Ingenuity Pathway Analysis (IPA).** List eQG consist of genes that resulted from combining of the differentially regulated genes on the microarray (n = 418) with marker positions from a study of quantitative trait loci (QTL) affecting susceptibility to mastitis in Norwegian Red cattle [26]. Red denotes molecules that were up-regulated in response to 6 hours infection with live *Staphylococcus aureus* in bovine macrophages.

Top networks mainly associated with cell-to-cell signaling and interaction, cell-mediated immune response and antimicrobial response were identified in IPA for the differentially expressed genes on microarray, while cell death and survival, cellular movement and immune cell trafficking were identified for List eQG (Table 4). All networks identified in IPA for both datasets are presented in Additional file 4: Table S4. The most striking functional network found to be over-represented in differentially expressed genes on the microarray involved *NFKB* and its associated molecules (Figure 2). For List eQG over-represented functional network involved *IL-4*, *IL-13*, *suppressor of cytokine signalling 3* (*SOCS3*) and its associated molecules (Figure 3).

**Table 4:** Top networks identified by Ingenuity Pathway Analysis (IPA)

	**Associated network functions**	**Score**
**A**	Cell-to-cell signaling and interaction, cardiovascular system development and function, cell morphology	30
	Embryonic development, organismal development, cell morphology	27
	Cell-mediated immune response, cellular movement, hematological system development and function	26
	Antimicrobial response, cell-to-cell signaling and interaction, embryonic development	23
	Cell cycle, cellular movement, developmental disorder	23
**B**	Cell death and survival, cellular function and maintenance, cellular growth and proliferation	20
	Cellular movement, hematological system development and function, immune cell trafficking	14
	Cellular assembly and organization, cellular compromise, cellular function and maintenance	8
	Antimicrobial response, cell-to-cell signaling and interaction, cellular movement	5
	Reproductive system disease, embryonic development, organismal development	3

A) The list of differentially regulated on microarray (n = 418) and Ensembl reannotated genes was used as an input for IPA; B) Subset of these genes (n = 28; List eQG from [18]) was used as an input.

**Figure 4:**
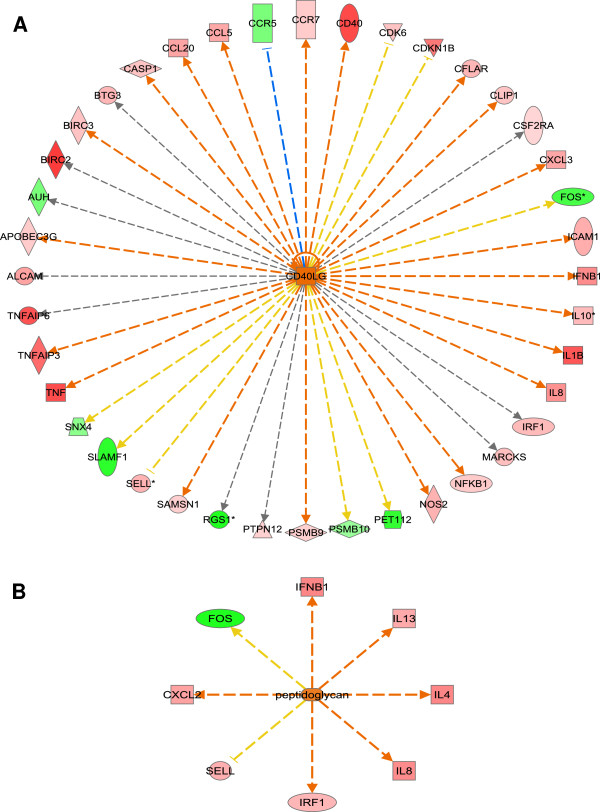
**Upstream regulators and their target molecules in datasets as identified by Ingenuity Pathway Analysis (IPA). A** – The list of differentially regulated on microarray (n = 418) and Ensembl reannotated genes was used as an input for analysis; **B** – Subset of these genes (n = 28; List eQG from [18]) was used as an input. Molecules in red denote up-regulation, molecules in green denote down-regulation and molecules in orange denote predicted activation in response to 6 hours infection with live *Staphylococcus aureus* in bovine macrophages. Lines in orange denote predicted activation; lines in blue - predicted inhibition; lines in yellow - findings inconsistent with state of downstream molecule; and lines in grey - effect not predicted.

The top upstream regulator identified in IPA for differentially expressed genes on the microarray was CD154 and for List eQG it was peptidoglycan. The top upstream regulators for both datasets are presented in Table 5 and all identified upstream regulators are presented in Additional file 5: Table S5. CD154 and peptidoglycan, and its target molecules in datasets are presented in Figure 4A and B, respectively.

**Table 5:** Top upstream regulators identified by Ingenuity Pathway Analysis (IPA)

	**Top upstream regulators**	***p*-value**
**A**	CD40 ligand (CD154)	2.25 × 10^-24^
	TNF receptor superfamily member 5 (CD40)	1.05 × 10^-22^
	Interleukin 10 (IL-10)	1.01 × 10^-21^
	5′-inosinic acid (poly rI:rC)	1.73 × 10^-20^
	Lipopolysaccharide (LPS)	6.21 × 10^-20^
**B**	Peptidoglycan	1.79 × 10^-13^
	Suppressor of cytokine signaling 1 (SOCS1)	7.66 × 10^-13^
	Tyrphostin AG490 (AG490)	1.71 × 10^-12^
	Interleukin 10 (IL-10)	3.52 × 10^-12^
	Toll-like receptor 2 (TLR2)	3.55 × 10^-12^

A) The list of differentially regulated on microarray (n = 418) and Ensembl reannotated genes was used as an input for IPA; B) Subset of these genes (n = 28; List eQG from [18]) was used as an input.

### Verification of microarray data by RT-qPCR

**Figure 5:**
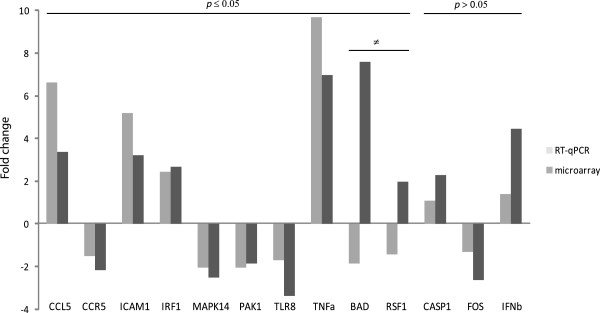
**Comparison of mRNA gene expression between the microarray and reverse transcription-quantitative PCR (RT-qPCR).** Genes that have shown a significant difference in expression between *Staphylococcus aureus* infected cells and uninfected control cells in RT-qPCR analysis are denoted with *p* ≤ 0.05 and non-significant genes in RT-qPCR are denoted with *p* > 0.05. Genes that have shown a divergent expression pattern in the RT-qPCR analysis compared to the microarray experiment are denoted with ≠. *Bcl2-antagonist of cell death* (*BAD*), *caspase 1* (*CASP1*), *chemokine c-c motif ligand 5* (*CCL5*), *chemokine c-c motif receptor 5* (*CCR5*), *v-fos fbj murine osteosarcoma viral oncogene homolog* (*FOS*), *intercellular adhesion molecule-1* (*ICAM1*), interferon beta (*IFNb*), *interferon regulatory factor 1* (*IRF1*), *mitogen-activated protein kinase 14, p38* (*MAPK14*), *p21/cdc42/rac1-activated kinase 1* (*PAK1*), *remodeling and spacing factor 1* (*RSF1*), *Toll-like receptor 8* (*TLR8*) and *tumor necrosis factor alpha* (*TNFa*). *Peptidylprolyl isomerase A* (*PPIA*) was used as a reference gene.

Ten out of thirteen genes selected for verification of the microarray results showed a significant difference in expression between *S. aureus* infected and uninfected control cells (*p* ≤ 0.05). Eight of these differentially expressed genes confirmed the expression pattern on the microarray [*chemokine c-c motif ligand 5* (*CCL5*), *chemokine c-c motif receptor 5* (*CCR5*), *intercellular adhesion molecule-1* (*ICAM1*), *interferon regulatory factor 1* (*IRF1*), *mitogen-activated protein kinase 14, p38* (*MAPK14*), *p21/cdc42/rac1-activated kinase 1* (*PAK1*), *TLR8* and *TNFa*], and two of the genes [*bcl2-antagonist of cell death* (*BAD*), and *remodeling and spacing factor 1* (*RSF1*)] showed a divergent expression pattern in the RT-qPCR analysis compared to the microarray experiment. *Caspase 1* (*CASP1*), *v-fos fbj murine osteosarcoma viral oncogene homolog* (*FOS*) and *IFNb* were non-significant in the RT-qPCR analysis, i.e. no significant difference in expression was observed between *S. aureus* infected and uninfected control individuals (*p* > 0.05; Figure 5). Overall, the RT-qPCR data correlated with the microarray data, 10 significant genes were used in the analyses (r = 0.6).

## Discussion

In this study we have investigated gene response of bovine macrophages to *in vitro* infection with live *S. aureus* in order to illuminate the putative mechanism underlying the early immune response for instance during mastitis in cattle. Our results have shown that infection of macrophages with *S. aureus* induced both alternative and classical activation pathways. The most pronounced functional network over-represented in the QTL related subset of our data (List eQG) involved anti-inflammatory cytokines *IL-4*, *IL-13* and *SOCS3* as central molecules and these genes were found to be up-regulated (Figure 3, Table 2B, Additional file 1: Table S1, Additional file 3: Table S3). Moreover, several proinflammatory cytokines classically activated upon TLR2 signaling in response to *S. aureus* were up-regulated in our study (Additional file 1: Table S1), e.g. *IL-1b* and *TNFa*, and TLR2 was identified as one of the putative upstream regulators (Table 5, Additional file 5: Table S5). It has been shown that TLR2 is involved in prolonged survival of *S. aureus* in macrophage phagosomes, which was explained by TLR2-dependent inhibition of superoxide production [19]. Our data support this study, however we suggest that *S. aureus* infection induces TLR2-dependent alternative activation of macrophages. This is consistent with the model proposed for intracellular bacterial survival of *F. tularensis. F. tularensis* enters macrophages by a TLR2-dependent mechanism that initiates a temporary inflammatory response. The macrophages are induced to produce IL-4 and IL-13 that in turn triggers alternative activation of macrophages and allows *F. tularensis* to replicate freely. Alternatively activated macrophages produce anti-inflammatory IL-10, which inhibits pro-inflammatory cytokines produced by classically activated macrophages [8]. The up-regulation of T helper 2 type cytokines *IL-4*, *IL-13* and *IL-10* (Additional file 1: Table S1) in our study may suggest that *S. aureus* infection has polarized bovine macrophages toward a M2 subset with wound repair M2a and immunoregulatory and anti-inflammatory M2c phenotypes.

Also, it has been demonstrated recently that infection with intracellular *Mycobacterium tuberculosis* activates the alternative phenotype of macrophages that inhibit autophagy-dependent maturation and killing of mycobacteria, which indicates that alternative activation of macrophages may contribute to the persistence of mycobacterial infection [20]. Furthermore, increased cell survival and viability with several inhibitors of apoptosis was found in our study as one of the top molecular functions and networks (Tables 3 and 4, Additional file 3: Table S3, Additional file 4: Table S4). *BAD*, *baculoviral IAP repeat-containing 2* (*BIRC2*), *BIRC3* and *BIRC5* were found to be up-regulated in response to *S. aureus* infection (Additional file 1: Table S1) and *BIRC5* was found among the differentially expressed genes in List eQG (Additional file 3: Table S3). It has been shown that phosphorylation of BAD protein and expression of *BIRC5* mRNA increased cell survival [21,22]. Moreover, it has been demonstrated that apoptosis in phagocytes can also be modulated by pathogens [23,24] and the increased expression of anti-apoptotic genes in *S. aureus* infected human macrophages is responsible for extended phagocyte lifetime allowing intracellular bacterial survival [25]. Interestingly, *BIRC5* and *SOCS3* are located in the region of QTL markers associated with somatic cell score (SCS) in NRF [26], and elevated SCS is often an indicator of subclinical mastitis in cattle infected with *S. aureus*. Likely, *S. aureus* evades the host immune system by induction of anti-inflammatory and anti-apoptotic factors in invaded host cells, which allows the pathogen to persist and replicate in the infected cells during the early stage of inflammation. These findings point toward *IL-4* and *IL-13* as possible expression QTL (eQTL) for *S. aureus* induced mastitis, and *SOCS3* and *BIRC5* as possible eQTL for chronic infections in cattle.

*CD40* and several proinflammatory cytokines e.g. *IL-1b*, *TNFa* and *IL-8* were up-regulated in our dataset, and CD154 was predicted as the most significant upstream regulator (Table 5, Figure 4). Recent studies have shown, that the synergy of TLR2 and CD40 signaling contributes to activation of resting B cells [27] and TLR2 signaling controls an early *Listeria monocytogenes* infection which involve co-stimulatory molecule CD40 [28]. Moreover, TLR2-dependent activation of NFKB complex and TNFa likely induced co-stimulatory function of CD40 signaling in the absence of other immune activators, such as T helper cells or IFNg [10,12,13]. This may suggest that TLR2-dependent activation may also require co-stimulatory function of CD40 for efficient *S. aureus* lipoproteins-induced signaling in macrophages. It has also been shown that CD40 signaling in macrophages is modulated by IL-4 and IL-10 anti-inflammatory cytokines that act via different mechanisms likely including SOCS [29,30], and IL-10 signaling was found as a top canonical pathway in our dataset (Table 2). This data together with findings that induction of *IL-10* expression in alternatively activated macrophages is TLR2-dependent suggest that CD40 signaling may likely be controlled by anti-inflammatory output of alternative activation of bovine macrophages induced by *S. aureus* infection.

The top KEGG pathway found in DAVID was the nucleotide-binding and oligomerization domain-like receptor (NLR) signaling pathway (Table 1), while the top canonical pathway found in IPA was triggering receptor expressed on myeloid cells 1 (TREM1) signaling (Table 2). The synergy between TLR2 and TREM1 stimulates intracellular signals resulting in phagocytosis and production of proinflammatory cytokines. Additionally, TREM1 signaling induces proinflammatory cytokines in response to bacterial muropeptides via the CASP1-dependent pathway. This signaling involves increased *NOD2* gene transcription suggesting that TREM1 acts to amplify the signal also from intracellular NLR proteins [31]. In addition to *TNFa* and *IL-1b*, *CASP1* and *NFKB1* were found to be up-regulated in our study (Additional file 1: Table S1) suggesting that TREM1 signaling may likely provide the link between TLR2- and NLR-mediated signal transduction in cytokine-induced inflammation during the initiation of host defense such as in mastitis. Furthermore, the most pronounced functional network over-represented in our data involved NFKB as a central molecule (Figure 2), suggesting that NFKB is a fundamental transcription factor linking TLR2-, CD40- and NLR-mediated signaling and induction of proinflammatory activities of macrophages in response to *S. aureus*.

Peptidoglycan was identified as the most significant upstream regulator for the List eQG (Table 5, Figure 4). Previous studies have shown that *S. aureus* lipoteichoic acid (LTA) and/or peptidoglycan activate the macrophages through intracellular NOD signaling [25,32]. It was demonstrated in cattle that the *S. aureus* peptidoglycan component synergized with LTA induced production of chemokines in mammary epithelial cells and neutrophil recruitment in the mammary gland [33]. This is also pronounced in our data, where the top canonical pathway for genes from List eQG was related to ‘Role of cytokines in mediating communication between immune cells’, with *IL-8* and *CXC-chemokine ligand 2* (*CXCL2*) chemokines among the significantly up-regulated genes (Table 2B, Figure 4B). These results suggest that *IL-8* and *CXCL2* may be potential eQTL for neutrophil recruitment during *S. aureus* induced intramammary infections in cattle. These findings together imply an important role for staphylococcal peptidoglycan in NOD signaling mediated immune response during mastitis in cattle with likely the principal role of neutrophil recruitment, as demonstrated earlier in the mammary gland [34-36]. Intracellular NLR are likely involved in peptidoglycan-induced signaling in macrophages, independent of lipopeptides/TLR2 interaction and signaling as demonstrated earlier [37].

We were not able to identify any significantly differentially expressed genes at the 2 h time point, while several genes were found at 3 h p.i in another study [38]. The variation between the individuals in macrophage gene expression in response to *S. aureus* infection was high at the 2 h time point resulting in the lack of significantly regulated genes. This may be due to relatively low expression level of immune response genes at its earliest stage of bacterial infection when high variation is more pronounced compared to the 6 h time point.

**Figure 6:**
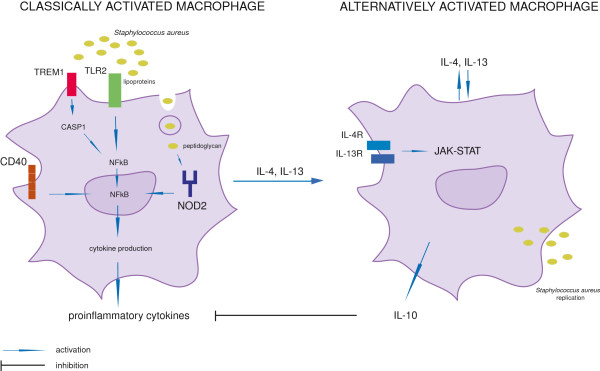
**Hypothetical mechanism of alternative activation of macrophages in response to *Staphylococcus aureus *infection.** Left panel – *Staphylococcus aureus* enters the macrophage through the Toll-like receptor 2 (TLR2)-dependent pathway that initiates nuclear factor kappa B (NFKB)-mediated temporal inflammatory response. Triggering receptor expressed on myeloid cells 1 (TREM1) synergizes with TLR2 that stimulates intracellular signals resulting in phagocytosis and production of proinflammatory cytokines. NFKB induces expression of co-stimulatory receptor tumor necrosis factor superfamily member 5 (CD40). After phagosomal escape into the cytosol *Staphylococcus aureus* peptidoglycan induces nucleotide-binding and oligomerization domain 2 (*NOD2*) expression that in turn triggers inflammation. The macrophage is induced to produce interleukin 4 (IL-4) and IL-13 as confirmed by our study. Right panel – hypothetical alternative activation pathway triggered by IL-4 and IL-13, likely a mechanism by which *Staphylococcus aureus* evades the host immune response. Alternatively activated macrophage produces anti-inflammatory IL-10, which inhibits classical macrophage activation. Caspase 1(CASP1); interleukin 4 receptor (IL-4R); interleukin 13 receptor (IL-13R); Janus kinase (JAK) and signal transducer and activator of transcription (STAT).

Our results are in agreement with previous studies of *S. aureus* mastitis in ruminants [38-43]. However, we have identified the anti-inflammatory cytokines *IL-4* and *IL-13*, which suggests that *S. aureus* infection probably leads to induction of alternative activation of the macrophages (Figure 6). The results of this study may reflect the importance of macrophages in modulating the host immune response during mastitis in cattle. It should be noted however, that our data are based on *in vitro* analysis of blood monocyte-derived macrophages. Consequently, the hypothesis needs to be further evaluated by studies of macrophages from bovine mammary glands infected with *S. aureus.*

## Conclusions

This study has demonstrated the novel finding that *S. aureus* infection most probably induces TLR2-dependent alternative activation of macrophages which contributes to persisting staphylococcal infection. Moreover, CD40 and TREM1 signaling pathways appear as the most interesting candidates that likely provide the link between TLR2- and NLR-mediated signal transduction determining optimal host defence during mastitis. Our study may reflect the role of macrophages in the immune defense of the udder and contributes to further elucidation of the mechanism of early inflammatory responses such as during mastitis in cattle.

## Methods

### Animals and cell isolation

Blood sampling was performed by a certified veterinarian and thus conducted in agreement with the provisions enforced by the Norwegian Animal Research Authority.

Twelve healthy and non-pregnant heifers of NRF at the age of 2–3 years were used in this study, six for the microarray experiment and six for validation of microarray results by RT-qPCR. Blood samples of 200 ml were collected from the neck of each animal in sterile glass bottles with 5 mM EDTA as anticoagulant. Peripheral blood mononuclear cells (PBMC) were extracted by gradient density centrifugation using Lymphoprep at 1600 × g (Axis-Shield, Kimbolton, UK). The number and viability of the PBMC were evaluated by Countess^®^ Automated Cell Counter (Life Technologies, Carlsbad, CA) and varied between 1.2 × 10^8^ - 6.6 × 10^8^ living cells.

Monocytes were extracted from the PBMC by positive selection of monocyte differentiation antigen CD14+ cells using anti human CD14 MACS MicroBeads (Miltenyi Biotec GmbH, Bergisch Gladbach, Germany) as described by the supplier. Purity of the selected cells was checked by flow cytometry (FACSCalibur) by staining selected cells that had already bound antiCD14 MACS beads with PE conjugated anti-mouse IgG1 (Southern Biotech, Birmingham, AL, USA; 1:200), and was in the range of 95-98%. CD14+ cells were grown in 6-well dishes at a density of 1.5 × 10^6^ - 2 × 10^6^ cells per well in a RPMI medium supplemented with 1 mM sodium pyruvate, 1 mM non-essential amino acids, 50 μM 2-mercaptoethanol and 10% FCS (all Invitrogen, Carlsbad, USA). The number of wells per individual varied between 12 and 24.

Cells were left over night at 37°C in an atmosphere with 5% CO_2_ to differentiate into macrophages and to minimize possible gene expression changes associated with cell stress caused by the isolation procedure. The phenotypic morphology of cells, i.e. differentiation of monocytes into adherent macrophages was visualized and confirmed by phase contrast microscopy.

### Bacterial infection

*S. aureus* strain 1685–4 isolated from a cow with clinical mastitis was used in the experiment [44]. This strain is susceptible to streptomycin and penicillin. Bacteria were grown in LB broth until mid-log phase. Growth was measured by optical density (OD) at 600 nm and the final number of colony-forming units (CFU) was determined by serial dilutions and plating on agar. Bacteria used in this study came from aliquots of the same batch.

For each individual the available number of wells with macrophages were grouped into four classes with equal number of wells and cells per class. Two classes were infected with *S. aureus* in a multiplicity of infection (MOI) of 1 (1 bacterium per cell, on average). The two other cell classes were left uninfected as controls.

After 1 h of infection, the wells were washed twice with PBS and new media containing 60 pg/ml penicillin and 100 μg/ml streptomycin was added to prevent growth of remaining extracellular bacteria. The control and the infected cells were treated equally. Inhibition of the bacterial growth by antibiotics was verified by microscopy.

Incubation was continued for one additional hour for one of the infected/control class of cells, a total of 2 h incubation, and for 5 more hours for the other class, a total of 6 h incubation. Media was aspirated and cells were frozen in the wells and stored at -80°C.

### RNA extraction

For microarray experiments, total RNA was extracted by a double extraction protocol. Cells were lysed directly in the wells using Trizol (Invitrogen) according to the manufacturer’s protocol. RNA extraction by Trizol was followed by RNA cleanup using RNeasy columns (Qiagen, Hilden, Germany). For RT-qPCR total RNA was extracted using the RNeasy kit (Qiagen) according to the manufacturer’s instructions, including on column DNase treatment.

RNA concentration and quality was measured using NanoDrop 1000 (Thermo Fisher Scientific, Wilmington, USA) and 2100 BioAnalyzer (Agilent Technologies, Palo Alto, USA), respectively. All samples used had a RNA integrity number (RIN) above 8 and OD A260/A280 ratio of approximately 2.

### Microarray experiment

Samples from six heifers (A-F) were used in the microarray experiment.

The microarray used was a 17 K bovine cDNA microarray constructed at ARK Genomics (ArrayExpress accession number A-MEXP1592). This array is an expansion of the bovine macrophage (BoMP) specific cDNA array focusing on immune response [45], including a BoMP library generated from stimulated bovine myeloid cells.

Due to the limited amount of RNA available, all samples were amplified using MessageAmp aRNA kit (Ambion, Austin, USA), including incorporation of aminoallyl UTP into the amplified RNA. Cy3 and Cy5 labeling of the aRNA was performed using either Cy3 or Cy5 Maleimide mono reactive dye (GE Healthcare Life Sciences, Buckinghamshire, UK) according to manufacturer’s instructions, followed by a cleanup procedure using DyeEx 2.0 spin kit (Qiagen).

The *S. aureus* infected samples were labeled with Cy3, and their respective uninfected control sample from one individual at one time point was labeled with Cy5 and hybridized together to one array. Dye-swap hybridization was performed, i.e. *S. aureus* infected sample was labeled with Cy5 and the respective uninfected control sample was labeled with Cy3 and hybridized together to one array. Although a full dye-swap hybridization design for all individuals was preferable [i.e. two arrays per each individual (A-F) per each time point (2 and 6 h)], the amount of RNA limited the number of dye swaps, resulting in a total of 19 arrays. See Table 6 for the details of dye-swap hybridization and Additional file 6: Figure S1 for experimental design.

**Table 6:** Microarray hybridization design

	**No of arrays/dye-swaps**						
Individual	A	B	C	D	E	F	Total
2 h incubation	2	1	1	1	1	2	8
6 h incubation	2	2	1	2	2	2	11

Uninfected control samples were used as references, i.e. control and *Staphylococcus aureus* infected samples from one individual at one time point were hybridized to one array. Dye-swap design was used, i.e. uninfected control samples were labeled with Cy5 and *Staphylococcus aureus* infected samples were labeled with Cy3, and subsequently swap labeling was applied.

Hybridizations were carried out in a GeneTac automated hybridization station (Genomic Solutions, Huntingdon, UK). Post hybridization the microarray slides were washed in wash buffers, post wash buffers and isopropanol. Dried slides were scanned in a Scanarray 5000 XL scanner (GSI Lumonics, Watertown, USA). Images were analyzed using BlueFuse for microarrays (BlueGenome, Cambridge, UK). Spots with poor confidence information, i.e. false discovery rate (FDR) ≥ 0.05, were removed from the analysis.

### Microarray data analysis

Normalization and analysis was performed using the Linear Models for Microarray Analysis (LIMMA) package of Bioconductor [46]. The normalized log ratios for each gene in each of the arrays were analyzed by split-plot ANOVA. The statistical cutoff value was set at FDR ≤ 0.05. Fold change ratios of gene expression comparisons were calculated as a mean of duplicate spots. All arrays (19) were included in these analyses.

The regulated probes (FDR ≤ 0.05) were reannotated to transform probe IDs into gene IDs recognized by pathway analysis software. Annotation was performed by identifying accession numbers in the annotated Ensembl bovine genomic database (ver. 52), by using blastn of fasta sequences against the Ensembl bovine transcripts (blast cutoff e-10) and if still unmatched, to the complete RefSeq RNA database at NCBI (http://www.ncbi.nlm.nih.gov/projects/RefSeq, blast cutoff e-5) as described by [47].

### RT-qPCR

Verification of differential gene expression was performed by RT-qPCR. Six heifers (G-L), additional to the ones used in the microarray experiment were used. Additional individuals were used as no RNA remained after microarray hybridization. Using new samples allows biological verification of the reproducibility of expression data (Additional file 6: Figure S1). Further, 100 ng of total RNA was reverse transcribed, cDNA was then diluted and 10 ng was used for qPCR. First strand cDNA synthesis was performed in triplicate (technical replicates) per sample using SuperScript III Reverse Transcriptase (Invitrogen). qPCR was performed using Power SYBR^®^ Green PCR Master Mix (Applied Biosystems, Valencia, USA) according to the manufacturer’s recommendations using 20 μl reaction volumes. Transcript levels were analyzed using a 7900HT Fast Real-Time PCR System (Applied Biosystems) according to the standard settings of the system software. Thirteen genes were chosen for RT-qPCR analysis, based on their regulation pattern in microarrays, affiliation to selected differentially regulated pathways and their putative role in mastitis pathogenesis based on literature. The genes are *BAD*, *CASP1*, *CCL5*, *CCR5*, *FOS*, *ICAM1*, *IFNb*, *IRF1*, *MAPK14*, *PAK1*, *RSF1*, *TLR8* and *TNFa*. Primers were designed using PrimerExpress ver. 1.5 (Applied Biosystems). Primer sequences are presented in Additional file 7: Table S6. A negative control for each RNA sample underwent the cDNA synthesis procedure without reverse transcriptase (no RT control), and negative controls with no added template were included for all primer pairs (no template control). The stability of six reference gene candidates (*ATP5B*, *EIF2B2I, GAPD*, *PPIA*, *RPL12I* and *SDHA*) was tested using the bovine geNorm house-keeping gene selection kit (PrimerDesign Ltd, Southampton, UK). Based on the geNorm results peptidylprolyl isomerase A (*PPIA*) was used as a single reference gene.

Uninfected samples were used as control and *S. aureus* infected samples were used as treatment. Fold change for each gene was calculated according to the formula: ${\text{Fold change}}_{\text{gene}\;x}=\frac{\Delta \mathrm{Ct}\;\text{treatment}}{\Delta \mathrm{\Delta Ct}\;\text{control}}$. Both ΔCt treatment and ΔCt control were averaged for the six animals prior to calculation of the fold change, and each animal was treated as a biological replicate. To facilitate interpretation of the down-regulated genes, the reciprocal and multiplied by -1 value of fold change was used. Only 6 h time point of infection was performed and analyzed.

Initial analysis of the RT-qPCR data was performed using RQ Manager 1.2 (Applied Biosystems). Standard deviation of ≤ 0.3 per triplicate was accepted. Due to the relatively small sample size in the experiment, a Wilcoxon signed-ranks test was applied for the RT-qPCR data analyses of control and treatment. *p*-values ≤ 0.05 were considered statistically significant. The statistical software InStat ver. 3 (GraphPad Software, San Diego, USA) was used for the analyses. Correlation analysis between RT-qPCR and microarray data was performed in Microsoft Excel.

### Pathway and cluster analyses

The list of differentially regulated on microarray (n = 418) and Ensembl reannotated genes was used as an input for pathway and cluster analyses. Moreover, a subset of these genes (n = 28; List eQG from [18]) was also used as an input for pathway analyses. Briefly, the List eQG consist of genes that resulted from combining the differentially regulated genes on the microarray (n = 418) with marker positions from a study of QTL affecting susceptibility to mastitis in NRF [26].

DAVID Resources v. 6.7 ([48,49]http://david.abcc.ncifcrf.gov/) was used for initial analysis to identify pathways generated by functional annotation clustering of gene lists and visualize genes on KEGG pathway maps. *p*-values≤ 0.05 were considered as significant and no correction for multiple testing was applied. Multiple testing correction techniques are known as conservative approaches and applying them may lower the sensitivity of pathway discovery.

The list of differentially expressed genes on microarray (n = 418) and the List eQG with corresponding fold change ratios were analyzed using IPA (http://www.ingenuity.com). New analyses in IPA were performed for List eQG, due to database update since the last analyses in 2011 [18]. IPA is a curated database and web-based analysis system that identifies the most significant canonical pathways, gene networks, key biological processes and upstream regulators in a set of genes of interest. Each gene from each gene list was mapped to its corresponding human gene ID object in the Ingenuity Pathways Knowledge Base. The resulting lists of genes were used as an input to the IPA library. To comprehend the biological background, significantly associated canonical pathways, biological functions, gene networks and upstream regulators were identified. The significance was determined based on *p*-values calculated using Fisher’s exact test, and *p-*value cut-off was set at 0.05. To summarize the large amount of data generated by IPA, we focused on and discussed selected most affected (top) biological functions belonging to the subgroup ‘Molecular and cellular functions’, the five most affected (top) canonical pathways, top gene networks and top upstream regulators.

Clustering of arrays (11 from the 6 h time point) and regulated genes was performed using Cluster 3.0 [50]. The list of differentially expressed genes with their corresponding fold change ratios was used in the analyses. Agglomerative hierarchical clustering with uncentered correlation and average linkage was used to construct the dendrograms. Java TreeView [51] was used to visualize the dendrograms.

### Availability of supporting data

The microarray expression data supporting the results are available in the Array Express Archive with accession E-TABM-1133 (http://www.ebi.ac.uk/arrayexpress/experiments/E-TABM-1133/)

The subset of differentially regulated genes (List eQG) obtained by comparison with data from genome-wide association mapping in NRF cattle can be found in reference [18].

Additional data are included as supplementary material in this paper.

## Competing interests

The authors declare that they have no competing interests.

## Authors’ contributions

GMB participated in the design of the study, carried out the infection experiments, microarray and RT-qPCR and has been involved in data analyses and drafting of the manuscript. AMLS performed the pathway and cluster analyses and drafted the manuscript. AD participated in the microarray experiment and performed microarray data analyses. RT contributed to study design and microarray data analyses. AKS participated in study design, infection experiments, flow cytometric analyses and manuscript drafting. IO participated in study design, data analyses and manuscript drafting. All authors read and approved the final manuscript.

## Supplementary Material

Additional file 1: Table S1 Genes and probes (n = 418) differentially expressed in infected cells vs. uninfected control cells 6 h post infection with *Staphylococcus aureus*. The genes/probes are clustered and listed in the same order (top to bottom) as in the dendrogram of Figure 1. Asterisk (*) denotes genes used in reverse transcription-quantitative PCR (RT-qPCR) verification of the microarray results. Click here for file

Additional file 2: Table S2 Significant canonical pathways generated by Ingenuity Pathway Analysis (IPA). A) The list of differentially regulated on microarray (n = 418) and Ensembl reannotated genes was used as an input for IPA; B) Subset of these genes (n = 28; List eQG from Lewandowska-Sabat *et al*., *Anim Genet* 2012, **43:**793–799) was used as an input. Asterisk (*) denotes canonical pathways significant after the Benjamini–Hochberg multiple testing correction (*p* ≤ 0.05). Click here for file

Additional file 3: Table S3 Biological functions identified by Ingenuity Pathway Analysis (IPA). A) The list of differentially regulated on microarray (n = 418) and Ensembl reannotated genes was used as an input for IPA; B) Subset of these genes (n = 28; List eQG from Lewandowska-Sabat *et al*., *Anim Genet* 2012, **43:**793–799) was used as an input. Click here for file

Additional file 4: Table S4 Networks identified by Ingenuity Pathway Analysis (IPA). A) The list of differentially regulated on microarray (n = 418) and Ensembl reannotated genes was used as an input for IPA; B) Subset of these genes (n = 28; List eQG from Lewandowska-Sabat *et al*., *Anim Genet* 2012, **43:**793–799) was used as an input. Click here for file

Additional file 5: Table S5 Upstream regulators identified by Ingenuity Pathway Analysis (IPA). A) The list of differentially regulated on microarray (n = 418) and Ensembl reannotated genes was used as an input for IPA; B) Subset of these genes (n = 28; List eQG from Lewandowska-Sabat *et al*., *Anim Genet* 2012, **43:**793–799) was used as an input. Click here for file

Additional file 6: Figure S1 Experimental design of microarray and reverse transcription-quantitative PCR (RT-qPCR) experiments. Letters A-L represent individual heifers used in the experiments, i.e. six heifers were used in the microarray and additional six heifers were used in the RT-qPCR; *S. aureus*: sample where blood monocyte-derived macrophages were infected with live *Staphylococcus aureus in vitro*; Control: sample of uninfected blood monocyte-derived macrophages; 2 h – 2 hours infection with *S. aureus*; 6 h – 6 hours infection with *Staphylococcus aureus*. For details on experimental design see Methods. Click here for file

Additional file 7: Table S6 List of primers used for reverse transcription-quantitative PCR (RT-qPCR). Click here for file
