# Supplementary material for: The early phase transcriptome of bovine monocyte-derived macrophages infected with Staphylococcus aureus in vitro
Source: BMC Genomics. 2013 Dec 17;14:891. doi: 10.1186/1471-2164-14-891 (PMC3878444; doi:10.1186/1471-2164-14-891)
Supplement: Additional file 1: Table S1 — Genes and probes (n = 418) differentially expressed in infected cells vs. uninfected control cells 6 h post infection with Staphylococcus aureus. The genes/probes are clustered and listed in the same order (top to bottom) as in the dendrogram of Figure 1. Asterisk (*) denotes genes used in reverse transcription-quantitative PCR (RT-qPCR) verification of the microarray results. [file 1471-2164-14-891-S1.docx]

**Table S1**. Genes and probes (n = 418) differentially expressed in infected cells vs. uninfected control cells 6 h post infection with *Staphylococcus aureus*. The genes / probes are clustered and listed in the same order (top to bottom) as in the dendrogram of Fig. 1. Asterisk (*) denotes genes used in reverse transcription-quantitative PCR (RT-qPCR) verification of the microarray results.

| ARK clone | Fold Change | Gene symbol | Human Entrez Gene ID | Gene name |
| --- | --- | --- | --- | --- |
| Cluster 1 | | | | |
| BE666665 | 7.59 | *BAD | 572 | BCL2-associated agonist of cell death |
| AJ820464 | 6.13 | ZNF668 | 79759 | - |
| Ovine IL1-b | 6.57 | IL1B | 3553 | interleukin 1, beta |
| AJ814178 | 2.98 | EEF1A1 | 1915 | eukaryotic translation elongation factor 1 alpha 1 |
| AJ816944 | 1.78 | TESK1 | 7016 | testis-specific kinase 1 |
| CO873919 | 1.98 | CDC42 | 998 | - |
| AJ674897 | 4.27 | CTSK | 1513 | - |
| AJ820746 | 1.74 | TMEM164 | 84187 | transmembrane protein 164 |
| AJ815818 | 1.92 | APOBEC3F | 200316 | - |
| AJ820719 | 2.11 | - | - | - |
| AJ818298 | 3.03 | RINT1 | 60561 | RAD50 interactor 1 |
| AW660024 | 3.54 | CCL20 | 6364 | chemokine (C-C motif) ligand 20 |
| AJ819453 | 7.98 | UBE3A | 7337 | ubiquitin protein ligase E3A |
| AM031001 | 7.32 | TNIP3 | 79931 | TNFAIP3 interacting protein 3 |
| AJ817163 | 4.76 | G3BP1 | 10146 | GTPase activating protein (SH3 domain) binding protein 1 |
| AM028934 | 7.60 | BIRC2 | 330 | baculoviral IAP repeat-containing 2 |
| AJ815220 | 2.07 | ATP6AP1 | 537 | ATPase, H+ transporting, lysosomal accessory protein 1 |
| BE750096 | 5.80 | CX3CL1 | 6376 | - |
| CD40 | 7.05 | CD40 | 958 | CD40 molecule, TNF receptor superfamily member 5 |
| AJ815958 | 2.34 | PPIF | 10105 | peptidylprolyl isomerase F |
| CN821981 | 2.40 | - | - | - |
| AJ819680 | 11.21 | ALS2cr13 | 150864 (57679) | - |
| S0000049E8 | 3.32 | BIRC5 | 332 | baculoviral IAP repeat-containing 5 |
| AJ815248 | 2.89 | - | - | - |
| AJ815223 | 2.47 | LOC644556 | 644556 | similar to exocyst complex component 5 |
| AJ815473 | 2.59 | CD164 | 8763 | CD164 molecule, sialomucin |
| AJ817546 | 2.03 | SOCS3 | 9021 | suppressor of cytokine signaling 3 |
| BI898472 | 1.96 | PTGDS | 5730 | - |
| AJ669970 | 3.00 | SDC4 | 6385 | syndecan 4 |
| AJ816887 | 4.91 | CYP27B1 | 1594 | cytochrome P450, family 27, subfamily B, polypeptide 1 |
| AJ819514 | 2.47 | SCFD2 | 152579 | sec1 family domain containing 2 |
| AJ814336 | 2.51 | IL4R | 3566 | interleukin 4 receptor |
| AM017561 | 2.36 | SETMAR | 6419 | SET domain and mariner transposase fusion gene |
| CO894199 | 2.12 | LOC731069 | 731069 | small nucleolar RNA host gene 8 (non-protein coding) |
| CO883665 | 3.37 | GCHFR | 2644 | - |
| AJ688793 | 3.00 | NOS2A | 4843 | nitric oxide synthase 2, inducible |
| BI775604 | 9.98 | IER3 | 8870 | immediate early response 3 |
| AJ814072 | 3.20 | ARL4A | 10124 | ADP-ribosylation factor-like 4A |
| CO894825 | 3.05 | TMEM158 | 25907 | transmembrane protein 158 |
| CO893758 | 1.89 | pseudo | 643397 | - |
| CN824208 | 3.13 | TP53INP2 | 58476 | tumor protein p53 inducible nuclear protein 2 |
| CO894116 | 2.64 | TP53INP2 | 58476 | tumor protein p53 inducible nuclear protein 2 |
| AM032138 | 3.99 | SFTPB | 6439 | surfactant protein B |
| AJ817229 | 3.02 | SLC39A8 | 64116 | solute carrier family 39 (zinc transporter), member 8 |
| AJ815274 | 2.56 | WTAP | 9589 | Wilms tumor 1 associated protein |
| AJ820628 | 2.11 | - | - | - |
| CO888898 | 2.15 | OSCAR | 126014 | osteoclast associated, immunoglobulin-like receptor |
| AJ697555 | 2.08 | P4HA2 | 8974 | prolyl 4-hydroxylase, alpha polypeptide II |
| AJ815395 | 4.59 | TNFSF13B | 10673 | tumor necrosis factor (ligand) superfamily, member 13b |
| AJ815611 | 3.03 | - | - | - |
| AJ691036 | 6.76 | TNFAIP6 | 7130 | tumor necrosis factor, alpha-induced protein 6 |
| AJ817995 | 2.81 | SNW1 | 22938 | SNW domain containing 1 |
| AJ815670 | 1.87 | SAMSN1 | 64092 | SAM domain, SH3 domain and nuclear localization signals 1 |
| AJ820252 | 2.62 | - | - | - |
| AJ818577 | 2.00 | Gng10 | 114119 | guanine nucleotide binding protein (G protein), gamma 10 |
| CN824724 | 2.10 | ACTR3 | 10096 | ARP3 actin-related protein 3 homolog (yeast) |
| AJ816104 | 1.88 | GPD2 | 2820 | glycerol-3-phosphate dehydrogenase 2 (mitochondrial) |
| AM009306 | 1.88 | GNAI3 | 2773 | guanine nucleotide binding protein (G protein), alpha inhibiting activity polypeptide 3 |
| AJ813688 | 2.79 | LRP6 | 4040 | low density lipoprotein receptor-related protein 6 |
| AJ814468 | 3.44 | Cxcl2 | 20310 | - |
| AJ820580 | 2.40 | RASAL2 | 9462 | RAS protein activator like 2 |
| AJ677542 | 1.97 | IRAK3 | 11213 | interleukin-1 receptor-associated kinase 3 |
| Ovine IL-8 | 4.27 | IL8 | 3576 | interleukin 8 |
| AM030167 | 2.12 | PIK3AP1 | 118788 | phosphoinositide-3-kinase adaptor protein 1 |
| AJ675768 | 2.84 | BTG3 | 10950 | BTG family, member 3 |
| AJ821008 | 2.63 | - | - | - |
| AM036229 | 1.90 | FAM49A | 81553 | family with sequence similarity 49, member A |
| AJ814853 | 5.38 | - | - | - |
| CN823380 | 2.31 | IL10 | 3586 | interleukin 10 |
| CO880131 | 2.47 | MARCKS | 4082 | myristoylated alanine-rich protein kinase C substrate |
| CO894308 | 2.68 | *IRF1 | 3659 | interferon regulatory factor 1 |
| CO880174 | 6.51 | TNFRSF9 | 3604 | - |
| CO874272 | 2.19 | ACAT2 | 39 | - |
| AJ818159 | 1.97 | - | - | - |
| AJ816528 | 3.80 | DOCK4 | 9732 | dedicator of cytokinesis 4 |
| AJ820542 | 3.24 | GNPTAB | 79158 | N-acetylglucosamine-1-phosphate transferase, alpha and beta subunits |
| AJ815324 | 2.61 | DVL3 | 1857 | - |
| AJ819618 | 2.11 | - | - | - |
| Ovine TNFa | 6.96 | *TNF | 7124 | tumor necrosis factor (TNF superfamily, member 2) |
| AJ819540 | 2.83 | CFLAR | 8837 | CASP8 and FADD-like apoptosis regulator |
| AJ813462 | 5.79 | TNFAIP3 | 7128 | tumor necrosis factor, alpha-induced protein 3 |
| AJ813138 | 2.94 | RPAP2 | 79871 | RNA polymerase II associated protein 2 |
| AJ818506 | 5.28 | CDKN1B | 1027 | cyclin-dependent kinase inhibitor 1B (p27, Kip1) |
| AJ820463 | 2.11 | CETN2 | 1069 | centrin, EF-hand protein, 2 |
| BM255808 | 1.80 | PSMB9 | 5698 | proteasome (prosome, macropain) subunit, beta type, 9 (large multifunctional peptidase 2) |
| AJ819120 | 7.09 | NP_055812.1 | 23008 | kelch domain containing 10 |
| BM288107 | 2.30 | *CASP1 | 834 | caspase 1, apoptosis-related cysteine peptidase (interleukin 1, beta, convertase) |
| AJ814061 | 3.21 | GBP5 | 115362 | guanylate binding protein 5 |
| AJ815121 | 2.17 | B4GALT5 | 9334 | UDP-Gal:betaGlcNAc beta 1,4- galactosyltransferase, polypeptide 5 |
| S0000049G2 | 3.15 | IL13 | 3596 | interleukin 13 |
| AM006516 | 1.81 | ETF1 | 2107 | eukaryotic translation termination factor 1 |
| CO895492 | 1.63 | RABGEF1 | 27342 | RAB guanine nucleotide exchange factor (GEF) 1 |
| AJ672249 | 1.86 | UGCG | 7357 | UDP-glucose ceramide glucosyltransferase |
| AM028963 | 1.85 | - | - | - |
| BF655096 | 2.65 | MEF2C | 4208 | myocyte enhancer factor 2C |
| CO895184 | 1.76 | - | - | - |
| AJ814632 | 2.66 | IL10 | 3586 | interleukin 10 |
| AJ820749 | 2.30 | GGNBP2 | 79893 | gametogenetin binding protein 2 |
| CO884344 | 2.10 | SLC20A1 | 6574 | solute carrier family 20 (phosphate transporter), member 1 |
| CN823907 | 2.13 | IER3 | 8870 | immediate early response 3 |
| CO892090 | 2.19 | PIM3 | 415116 | pim-3 oncogene |
| CO879951 | 1.79 | UTX | 7403 | - |
| BF775518 | 2.22 | - | - | - |
| CO873582 | 1.76 | - | - | - |
| AM007975 | 1.87 | SRI | 6717 | sorcin |
| AJ689205 | 2.05 | - | 23382 | S-adenosylhomocysteine hydrolase-like 2 |
| CO879385 | 1.75 | ATP6V1C1 | 528 | ATPase, H+ transporting, lysosomal 42kDa, V1 subunit C1 |
| CO871952 | 2.67 | RAMP3 | 10268 | receptor (G protein-coupled) activity modifying protein 3 |
| AJ819834 | 5.35 | - | - | - |
| AM009614 | 2.54 | ACSL5 | 51703 | acyl-CoA synthetase long-chain family member 5 |
| C0005342G16 | 2.18 | ACSL5 | 51703 | acyl-CoA synthetase long-chain family member 5 |
| CO892004 | 2.18 | SLC27A1 | 376497 | solute carrier family 27 (fatty acid transporter), member 1 |
| CO892949 | 2.38 | FMNL3 | 91010 | formin-like 3 |
| CO890242 | 2.27 | - | - | - |
| C0005338K24 | 3.43 | EPB42 | 2038 | erythrocyte membrane protein band 4.2 |
| AM008924 | 3.60 | EPB42 | 2038 | erythrocyte membrane protein band 4.2 |
| CO883177 | 2.16 | - | - | - |
| AM020681 | 2.69 | CYP3A4 | 1576 | cytochrome P450, family 3, subfamily A, polypeptide 4 |
| CO893352 | 1.94 | - | - | - |
| CO893694 | 2.73 | - | - | - |
| CO883272 | 2.97 | SELL | 6402 | selectin L |
| CN822436 | 3.19 | - | - | - |
| CO893404 | 2.57 | TTC19 | 54902 | tetratricopeptide repeat domain 19 |
| CO876090 | 2.36 | - | - | - |
| CO890253 | 2.58 | - | - | - |
| CO885519 | 2.31 | - | - | - |
| CO895370 | 2.73 | - | - | - |
| CO894469 | 2.32 | - | - | - |
| CN821857 | 2.11 | - | - | - |
| CO890719 | 2.43 | - | - | - |
| CO893714 | 2.29 | GRIK2 | 2898 | glutamate receptor, ionotropic, kainate 2 |
| CO889996 | 2.18 | REPIN1 | 29803 | replication initiator 1 |
| AJ813979 | 2.67 | - | - | - |
| CO895016 | 2.04 | HSD17B10 | 3028 | - |
| AJ813088 | 3.13 | - | - | - |
| AJ814077 | 3.18 | C1orf21 | 81563 | chromosome 1 open reading frame 21 |
| CO889735 | 2.30 | LOC652727 | 652727 | - |
| AJ690407 | 2.53 | SRI | 6717 | sorcin |
| CO893307 | 2.84 | - | - | - |
| CO889173 | 2.70 | - | - | - |
| CN822718 | 2.75 | SFTPB | 6439 | surfactant protein B |
| CO893154 | 2.29 | NP_001073865.1 | 728215 | - |
| CO889752 | 2.32 | C9orf84 | 158401 | - |
| CCL5 | 3.37 | *CCL5 | 6352 | chemokine (C-C motif) ligand 5 |
| CO891746 | 2.53 | SAPS2 | 9701 | SAPS domain family, member 2 |
| CO895158 | 2.13 | JAZF1 | 221895 | JAZF zinc finger 1 |
| AJ818740 | 2.72 | - | - | - |
| CO892067 | 2.44 | - | - | - |
| AJ817981 | 2.93 | CIRBP | 1153 | cold inducible RNA binding protein |
| AJ819700 | 2.46 | MYL6 | 4637 | myosin, light chain 6, alkali, smooth muscle and non-muscle |
| CO877872 | 1.88 | MED15 | 51586 | mediator complex subunit 15 |
| CO883018 | 3.06 | - | - | - |
| CO889379 | 2.69 | SFTPB | 6439 | - |
| CO880468 | 1.94 | - | - | - |
| CO890198 | 3.05 | - | - | - |
| CO893731 | 2.23 | TEX2 | 55852 | testis expressed 2 |
| CO890011 | 2.04 | - | - | - |
| CO895561 | 2.56 | SELL | 6402 | selectin L |
| CO874479 | 2.25 | - | - | - |
| CO882903 | 2.26 | - | - | - |
| CO889280 | 2.14 | - | - | - |
| CN823917 | 1.81 | FAM82B | 51115 | family with sequence similarity 82, member B |
| CO891208 | 2.12 | MKKS | 8195 | McKusick-Kaufman syndrome |
| CO890847 | 2.30 | - | - | - |
| AJ815783 | 2.11 | - | - | - |
| CO883837 | 2.82 | ATP9A | 10079 | microfibrillar-associated protein 3-like |
| AJ819233 | 2.64 | - | - | - |
| CO896976 | 2.02 | TXNRD1 | 7296 | thioredoxin reductase 1 |
| AM029817 | 1.83 | ERBB2IP | 55914 | erbb2 interacting protein |
| AJ817409 | 2.32 | CDK6 | 1021 | cyclin-dependent kinase 6 |
| AJ818846 | 2.78 | - | - | - |
| CN823239 | 1.88 | - | - | - |
| AJ819705 | 2.03 | SEP11 | 55752 | septin 11 |
| CN822622 | 2.01 | PPAP2B | 8613 | phosphatidic acid phosphatase type 2B |
| AM037100 | 2.66 | NUPR1 | 26471 | nuclear protein, transcriptional regulator, 1 |
| CO887080 | 3.74 | C17orf28 | 283987 | chromosome 17 open reading frame 28 |
| AJ817839 | 2.48 | RDBP | 7936 | RD RNA binding protein |
| AM013662 | 3.20 | *ICAM1 | 3383 | intercellular adhesion molecule 1 |
| CN824022 | 5.42 | SGK1 | 6446 | serum/glucocorticoid regulated kinase 1 |
| AJ819780 | 1.79 | - | - | - |
| AJ814805 | 3.39 | CFB | 629 | complement factor B |
| AJ814908 | 2.34 | - | - | - |
| S0000049F9 | 4.47 | *IFNB1 | 3456 | interferon, beta 1, fibroblast |
| AJ815871 | 1.90 | ERC1 | 9588 | peroxiredoxin 6 |
| CN824006 | 1.85 | TUBA1A | 7846 | - |
| AM031455 | 1.85 | EMR1 | 2015 | egf-like module containing, mucin-like, hormone receptor-like 1 |
| AJ818568 | 2.78 | - | - | - |
| CO872874 | 2.01 | - | - | - |
| AJ815521 | 7.21 | pseudo | 391026 | - |
| CN824709 | 1.78 | PRDX1 | 5052 | peroxiredoxin 1 |
| AJ818180 | 2.12 | ISCA1 | 81689 | iron-sulfur cluster assembly 1 homolog (S. cerevisiae) |
| CO881606 | 2.16 | - | - | - |
| CO886922 | 1.96 | GRB2 | 2885 | growth factor receptor-bound protein 2 |
| CO890685 | 1.68 | GPC1 | 2817 | glypican 1 |
| CO893485 | 1.72 | MAOA | 4128 | monoamine oxidase A |
| CO202972 | 1.66 | - | - | - |
| CO879862 | 2.73 | MOBP | 4336 | myelin-associated oligodendrocyte basic protein |
| CO881495 | 2.11 | AMD1 | 262 | adenosylmethionine decarboxylase 1 |
| AJ814331 | 2.99 | SGMS2 | 166929 | sphingomyelin synthase 2 |
| AJ819076 | 3.65 | - | - | - |
| AJ819528 | 3.50 | POFUT1 | 23509 | protein O-fucosyltransferase 1 |
| AJ816613 | 3.14 | CHORDC1 | 26973 | cysteine and histidine-rich domain (CHORD)-containing 1 |
| AJ818078 | 2.47 | RB11 | 534712 | retinoblastoma 1 |
| AM015556 | 1.98 | UAP1L1 | 91373 | - |
| AM022587 | 2.34 | BIRC3 | 330 | baculoviral IAP repeat-containing 3 |
| AJ814138 | 2.27 | PCNA | 5111 | proliferating cell nuclear antigen |
| AJ821181 | 2.00 | - | - | - |
| AJ819363 | 3.28 | MAN2A1 | 4124 | mannosidase, alpha, class 2A, member 1 |
| AM018868 | 2.06 | NFKB1 | 4790 | - |
| BF652066 | 2.35 | IFNGR1 | 3459 | interferon gamma receptor 1 |
| AM032196 | 2.02 | APOBEC3G | 60489 | apolipoprotein B mRNA editing enzyme, catalytic polypeptide-like 3G |
| CO890427 | 1.92 | CLDND1 | 56650 | claudin domain containing 1 |
| AJ814609 | 3.57 | ALCAM | 214 | activated leukocyte cell adhesion molecule |
| AJ818308 | 1.87 | ERC1 | 23085 | ELKS/RAB6-interacting/CAST family member 1 |
| AJ816454 | 2.74 | - | - | - |
| AJ816971 | 2.16 | - | - | - |
| AJ817857 | 1.98 | CDKL2 | 8999 | cyclin-dependent kinase-like 2 (CDC2-related kinase) |
| CO882747 | 1.79 | PEX6 | 5190 | peroxisomal biogenesis factor 6 |
| CO876543 | 1.91 | - | - | - |
| AJ815407 | 1.86 | MAF | 4094 | v-maf musculoaponeurotic fibrosarcoma oncogene homolog (avian) |
| CO882425 | 1.81 | - | - | - |
| CO892589 | 1.78 | - | - | - |
| CO879247 | 1.68 | DPH3 | 285381 | DPH3, KTI11 homolog (S. cerevisiae) |
| AM018684 | 1.63 | CSF2RA | 1438 | colony stimulating factor 2 receptor, alpha, low-affinity (granulocyte-macrophage) |
| CO896740 | 1.81 | TUBA8 | 51807 | tubulin, alpha 8 |
| AM032025 | 3.81 | HECTD2 | 143279 | HECT domain containing 2 |
| AJ813932 | 2.15 | - | - | - |
| S0000049A3 | 4.48 | IL4 | 3565 | interleukin 4 |
| AJ815426 | 1.96 | - | - | - |
| AM018482 | 2.01 | THOC1 | 9984 | THO complex 1 |
| AM036412 | 1.71 | MRLC2 | 103910 | myosin, light chain 12B, regulatory |
| Ovine IL-10 | 2.47 | IL10 | 3586 | interleukin 10 |
| AJ818364 | 1.88 | DCP2 | 167227 | DCP2 decapping enzyme homolog (S. cerevisiae) |
| CO896781 | 2.95 | SLC11A2 | 4891 | solute carrier family 11 (proton-coupled divalent metal ion transporters), member 2 |
| CO887050 | 2.16 | AXL | 558 | AXL receptor tyrosine kinase |
| AJ819250 | 1.74 | FOXO1 | 2308 | forkhead box O1 |
| AJ818904 | 1.70 | HS2ST1 | 9653 | heparan sulfate 2-O-sulfotransferase 1 |
| CO888523 | 2.29 | - | - | - |
| CO891067 | 1.68 | RASSF3 | 283349 | - |
| BI541179 | 2.02 | CCR7 | 1236 | chemokine (C-C motif) receptor 7 |
| AJ820942 | 2.00 | - | - | - |
| AJ815464 | 1.97 | *RSF1 | 51773 | remodeling and spacing factor 1 |
| AJ817691 | 1.64 | UBE2W | 55284 | ubiquitin-conjugating enzyme E2W (putative) |
| AJ816876 | 1.82 | PTPN12 | 5782 | protein tyrosine phosphatase, non-receptor type 12 |
| AJ820847 | 3.40 | GPR155 | 151556 | G protein-coupled receptor 155 |
| AJ819997 | 2.23 | CLIP1 | 6249 | CAP-GLY domain containing linker protein 1 |
| AJ819567 | 2.56 | - | - | - |
| AJ820675 | 1.90 | - | - | - |
| AJ816402 | 1.63 | TFB2M | 64216 | transcription factor B2, mitochondrial |
| CO886677 | 1.91 | C17orf68 | 80169 | chromosome 17 open reading frame 68 |
| Cluster 2 | | | | |
| CO890973 | -1.66 | PSMB10 | 5699 | proteasome (prosome, macropain) subunit, beta type, 10 |
| AJ674105 | -1.70 | - | - | - |
| AJ817591 | -1.71 | PTPLAD1 | 51495 | protein tyrosine phosphatase-like A domain containing 1 |
| CO888188 | -1.81 | - | - | - |
| AM010765 | -1.67 | APPBP2 | 10513 | amyloid beta precursor protein (cytoplasmic tail) binding protein 2 |
| AJ820799 | -1.68 | - | - | - |
| AJ816282 | -1.68 | - | - | - |
| AJ819033 | -2.63 | - | - | - |
| AJ819925 | -2.17 | QRSL1 | 55278 | glutaminyl-tRNA synthase (glutamine-hydrolyzing)-like 1 |
| AJ813437 | -1.72 | ZFYVE16 | 9765 | zinc finger, FYVE domain containing 16 |
| AJ821024 | -2.33 | - | - | - |
| AJ819020 | -1.85 | DOCK11 | 139818 | dedicator of cytokinesis 11 |
| AJ816126 | -1.92 | FCGR2B | 2213 | Fc fragment of IgG, low affinity IIb, receptor (CD32) |
| AJ820285 | -1.95 | - | - | - |
| AJ821249 | -1.63 | pseudo | 391158 | - |
| AJ820826 | -1.74 | - | - | - |
| AJ813012 | -1.90 | - | - | - |
| AM022955 | -1.70 | CCDC125 | 202243 | coiled-coil domain containing 125 |
| CO888633 | -2.13 | PCID2 | 55795 | PCI domain containing 2 |
| AJ818715 | -1.80 | - | - | - |
| AM015680 | -2.81 | METTL7A | 25840 | methyltransferase like 7A |
| AM022337 | -1.87 | EIF3K | 27335 | eukaryotic translation initiation factor 3, subunit K |
| CO891778 | -1.67 | - | - | - |
| CO876584 | -1.83 | FOXN3 | 1112 | forkhead box N3 |
| CO894148 | -1.79 | ASAHL | 27163 | - |
| AJ814750 | -1.68 | TBL1X | 6907 | transducin (beta)-like 1X-linked |
| CO875299 | -1.77 | ERP29 | 10961 | endoplasmic reticulum protein 29 |
| AM024035 | -1.78 | - | - | - |
| CO880431 | -1.90 | SNHG8 | 100093630 | - |
| CO881956 | -1.72 | MAEA | 10296 | casein kinase 1, epsilon |
| AJ695385 | -1.92 | NCAPH | 23397 | - |
| CO877089 | -1.88 | - | - | - |
| CO879769 | -1.94 | - | - | - |
| AJ813550 | -2.01 | - | - | - |
| CO202587 | -1.93 | GLO1 | 2739 | glyoxalase I |
| CO872018 | -1.99 | FAM102B | 284611 | - |
| AJ675270 | -2.29 | NRG1 | 3084 | neuregulin 1 |
| AJ814091 | -2.08 | ALDH2 | 217 | aldehyde dehydrogenase 2 family (mitochondrial) |
| AJ690665 | -2.17 | *CCR5 | 1234 | chemokine (C-C motif) receptor 5 |
| AJ813201 | -2.41 | ARL4C | 10123 | ADP-ribosylation factor-like 4C |
| AM022656 | -1.88 | ZNF643 | 65243 | - |
| AM027039 | -2.21 | FCGR3A | 2214 | Fc fragment of IgG, low affinity IIIa, receptor (CD16a) |
| AM037006 | -1.75 | ETAA1 | 54465 | Ewing tumor-associated antigen 1 |
| AM017061 | -1.80 | F13B | 2165 | - |
| AJ817200 | -1.87 | - | - | - |
| AM009552 | -1.68 | TIM21 | 852921 | - |
| CN823289 | -2.27 | *FOS | 2353 | v-fos FBJ murine osteosarcoma viral oncogene homolog |
| AJ814124 | -1.69 | C3orf26 | 84319 | chromosome 3 open reading frame 26 |
| CN821785 | -1.72 | MAT2B | 27430 | methionine adenosyltransferase II, beta |
| AJ819330 | -1.82 | DPY30 | 84661 | dpy-30 homolog (C. elegans) |
| AJ819301 | -1.74 | - | - | - |
| AJ817904 | -2.10 | - | - | - |
| AJ673977 | -2.01 | ENPP1 | 5167 | ectonucleotide pyrophosphatase/phosphodiesterase 1 |
| AJ820736 | -2.18 | - | - | - |
| AJ814913 | -1.73 | - | - | - |
| AJ817288 | -1.67 | - | - | - |
| AJ817958 | -1.73 | ZNF655 | 79027 | zinc finger protein 655 |
| AJ818882 | -1.79 | - | - | - |
| AJ818489 | -2.10 | - | - | - |
| AJ819668 | -2.21 | - | - | - |
| AM015996 | -1.83 | - | - | - |
| AJ814016 | -1.88 | ZMYM6 | 9204 | zinc finger, MYM-type 6 |
| AJ816803 | -1.82 | FOXP1 | 27086 | forkhead box P1 |
| CO872792 | -1.85 | APPL2 | 55198 | adaptor protein, phosphotyrosine interaction, PH domain and leucine zipper containing 2 |
| AJ820100 | -1.98 | ERH | 2079 | enhancer of rudimentary homolog (Drosophila) |
| AJ819148 | -2.07 | - | - | - |
| AJ813048 | -1.89 | CRYZL1 | 9946 | crystallin, zeta (quinone reductase)-like 1 |
| AJ821000 | -1.86 | RASA2 | 5922 | RAS p21 protein activator 2 |
| AJ819763 | -1.79 | - | - | - |
| AJ820374 | -2.40 | ARRDC4 | 91947 | arrestin domain containing 4 |
| AJ815127 | -1.82 | PAPD4 | 167153 | PAP associated domain containing 4 |
| BF230296 | -1.98 | TRAF6 | 7189 | TNF receptor-associated factor 6 |
| AJ818147 | -1.69 | C13orf31 | 144811 | chromosome 13 open reading frame 31 |
| AJ819239 | -2.35 | - | - | - |
| AJ816590 | -2.95 | MSL3L1 | 10943 | male-specific lethal 3 homolog (Drosophila) |
| AJ817642 | -1.65 | - | - | - |
| AJ819756 | -1.89 | SNX4 | 8723 | sorting nexin 4 |
| AJ820897 | -1.77 | CCNDBP1 | 23582 | cyclin D-type binding-protein 1 |
| AJ671824 | -2.50 | CLEC7A | 64581 | C-type lectin domain family 7, member A |
| CO889725 | -2.20 | CNKSR1 | 10256 | connector enhancer of kinase suppressor of Ras 1 |
| AJ816331 | -2.60 | - | - | - |
| AJ816915 | -2.86 | - | - | - |
| CN824237 | -1.79 | Q6MZR2_HUMAN | - | - |
| AJ674659 | -2.09 | TNRC6B | 23112 | trinucleotide repeat containing 6B |
| AJ817616 | -2.18 | - | - | - |
| AJ675370 | -1.73 | h2afv | 252913 | - |
| AJ813203 | -2.00 | RARRES1 | 5918 | retinoic acid receptor responder (tazarotene induced) 1 |
| AJ813038 | -1.74 | DDEF2 | 8853 | ArfGAP with SH3 domain, ankyrin repeat and PH domain 2 |
| CO886445 | -1.87 | MERTK | 10461 | c-mer proto-oncogene tyrosine kinase |
| AJ818075 | -1.81 | - | - | - |
| AM028922 | -1.92 | CRLF3 | 51379 | cytokine receptor-like factor 3 |
| AJ817284 | -2.00 | LRRC8D | 55144 | leucine rich repeat containing 8 family, member D |
| AM039218 | -1.68 | ALDH9A1 | 223 | aldehyde dehydrogenase 9 family, member A1 |
| AM006448 | -2.10 | GFM1 | 85476 | G elongation factor, mitochondrial 1 |
| C0005604J6 | -1.98 | - | - | - |
| AJ818999 | -1.88 | - | - | - |
| AJ818946 | -2.06 | - | - | - |
| C0005604D2 | -1.85 | PBX3 | 5090 | pre-B-cell leukemia homeobox 3 |
| AJ819101 | -1.85 | - | - | - |
| AJ813676 | -1.79 | ZMYND11 | 10771 | zinc finger, MYND domain containing 11 |
| AJ671853 | -2.11 | TRIM6 | 117854 | - |
| AJ814299 | -1.70 | - | - | - |
| AM029883 | -2.24 | EIF2B5 | 8893 | - |
| CO873884 | -1.81 | - | - | - |
| CO888906 | -2.32 | ARL4C | 10123 | ADP-ribosylation factor-like 4C |
| AJ817831 | -1.98 | UNG | 7374 | uracil-DNA glycosylase |
| AJ819263 | -1.82 | LUZP1 | 7798 | leucine zipper protein 1 |
| CO892041 | -2.60 | KLF13 | 51621 | Kruppel-like factor 13 |
| AM039117 | -2.08 | CASC1 | 55259 | - |
| AJ672081 | -2.84 | ABCC1 | 4363 | - |
| AM031610 | -3.66 | PDK1 | 5163 | pyruvate dehydrogenase kinase, isozyme 1 |
| CO889703 | -1.67 | - | - | - |
| AJ818893 | -1.70 | - | - | - |
| AJ815443 | -1.77 | - | - | - |
| AJ817479 | -1.86 | TRAF3IP3 | 80342 | TRAF3 interacting protein 3 |
| AJ821081 | -1.88 | NFIB | 4781 | nuclear factor I/B |
| AJ695354 | -1.93 | KIAA0586 | 9786 | KIAA0586 |
| AJ812930 | -1.86 | GLRX | 2745 | glutaredoxin (thioltransferase) |
| AM039266 | -2.05 | CLEC12A | 160364 | - |
| AJ816119 | -4.34 | RDH11 | 51109 | - |
| AJ816942 | -2.14 | ANXA1 | 301 | annexin A1 |
| AJ815202 | -2.13 | CHORDC1 | 26973 | cysteine and histidine-rich domain (CHORD)-containing 1 |
| AJ815761 | -2.23 | KLF11 | 8462 | Kruppel-like factor 11 |
| AM013846 | -2.26 | LACTB | 114294 | lactamase, beta |
| AJ820142 | -2.18 | AUH | 549 | AU RNA binding protein/enoyl-Coenzyme A hydratase |
| AJ816372 | -2.57 | - | - | - |
| AJ814218 | -2.53 | *MAPK14 | 1432 | mitogen-activated protein kinase 14 |
| AJ814934 | -2.69 | UGT8 | 7368 | - |
| AJ819046 | -1.79 | - | - | - |
| AJ816629 | -2.20 | MTCH2 | 23788 | mitochondrial carrier homolog 2 (C. elegans) |
| AM029921 | -3.10 | RGS1 | 5996 | regulator of G-protein signaling 1 |
| C0005596M3 | -3.68 | RGS1 | 5996 | regulator of G-protein signaling 1 |
| AJ819035 | -3.92 | - | - | - |
| AM012392 | -2.43 | HNMT | 3176 | histamine N-methyltransferase |
| AM035763 | -2.24 | ANKRD29 | 147463 | ankyrin repeat domain 29 |
| AJ816550 | -3.53 | IL6R | 3570 | interleukin 6 receptor |
| AJ819990 | -2.22 | - | - | - |
| AJ815316 | -3.35 | PET112L | 5188 | PET112-like (yeast) |
| AJ815351 | -3.46 | SLAMF1 | 6504 | signaling lymphocytic activation molecule family member 1 |
| CN824048 | -2.64 | FOS | 2353 | v-fos FBJ murine osteosarcoma viral oncogene homolog |
| AM009178 | -3.01 | FOS | 2353 | v-fos FBJ murine osteosarcoma viral oncogene homolog |
| S0000049E10 | -3.32 | *TLR8 | 51311 | toll-like receptor 8 |
| AJ821185 | -2.74 | ACTR8 | 93973 | ARP8 actin-related protein 8 homolog (yeast) |
| AJ816784 | -2.07 | USP52 | 9924 | PAN2 poly(A) specific ribonuclease subunit homolog (S. cerevisiae) |
| AJ815315 | -2.42 | - | - | - |
| AJ819849 | -2.67 | - | - | - |
| CO887675 | -2.41 | CCDC6 | 8030 | coiled-coil domain containing 6 |
| CO202932 | -2.20 | - | - | - |
| AJ819478 | -3.66 | - | - | - |
| AM028095 | -1.86 | KBTBD3 | 143879 | kelch repeat and BTB (POZ) domain containing 3 |
| AJ814705 | -1.94 | - | - | - |
| AJ813066 | -2.09 | - | - | - |
| AJ814411 | -2.25 | ZEB2 | 9839 | zinc finger E-box binding homeobox 2 |
| AJ812769 | -2.47 | - | - | - |
| AJ817211 | -1.74 | - | 56731 | SLC2A4 regulator |
| AJ819525 | -2.45 | - | - | - |
| AJ816860 | -2.61 | - | - | - |
| AJ817298 | -2.13 | - | - | - |
| AJ814742 | -2.27 | EPB41 | 2035 | erythrocyte membrane protein band 4.1 (elliptocytosis 1, RH-linked) |
| AJ818971 | -2.14 | - | - | - |
| AJ813845 | -2.68 | - | - | - |
| CO889804 | -1.87 | *PAK1 | 5058 | p21 protein (Cdc42/Rac)-activated kinase 1 |
| AJ820868 | -2.48 | DUSP12 | 11266 | dual specificity phosphatase 12 |
| AJ815389 | -2.37 | - | - | - |
| AJ813595 | -2.73 | ZEB2 | 9839 | - |
| AJ816583 | -2.78 | PRKD3 | 23683 | protein kinase D3 |
| AJ815931 | -1.83 | Dtwd1 | 69185 | - |
| CN821814 | -2.53 | EGLN2 | 112398 | egl nine homolog 2 (C. elegans) |
