# Supplementary material for: The early phase transcriptome of bovine monocyte-derived macrophages infected with Staphylococcus aureus in vitro
Source: BMC Genomics. 2013 Dec 17;14:891. doi: 10.1186/1471-2164-14-891 (PMC3878444; doi:10.1186/1471-2164-14-891)
Supplement: Additional file 6: Figure S1 — Experimental design of microarray and reverse transcription-quantitative PCR (RT-qPCR) experiments. Letters A-L represent individual heifers used in the experiments, i.e. six heifers were used in the microarray and additional six heifers were used in the RT-qPCR; S. aureus: sample where blood monocyte-derived macrophages were infected with live Staphylococcus aureus in vitro; Control: sample of uninfected blood monocyte-derived macrophages; 2 h – 2 hours infection with S. aureus; 6 h – 6 hours infection with Staphylococcus aureus. For details on experimental design see Methods. [file 1471-2164-14-891-S6.pdf]

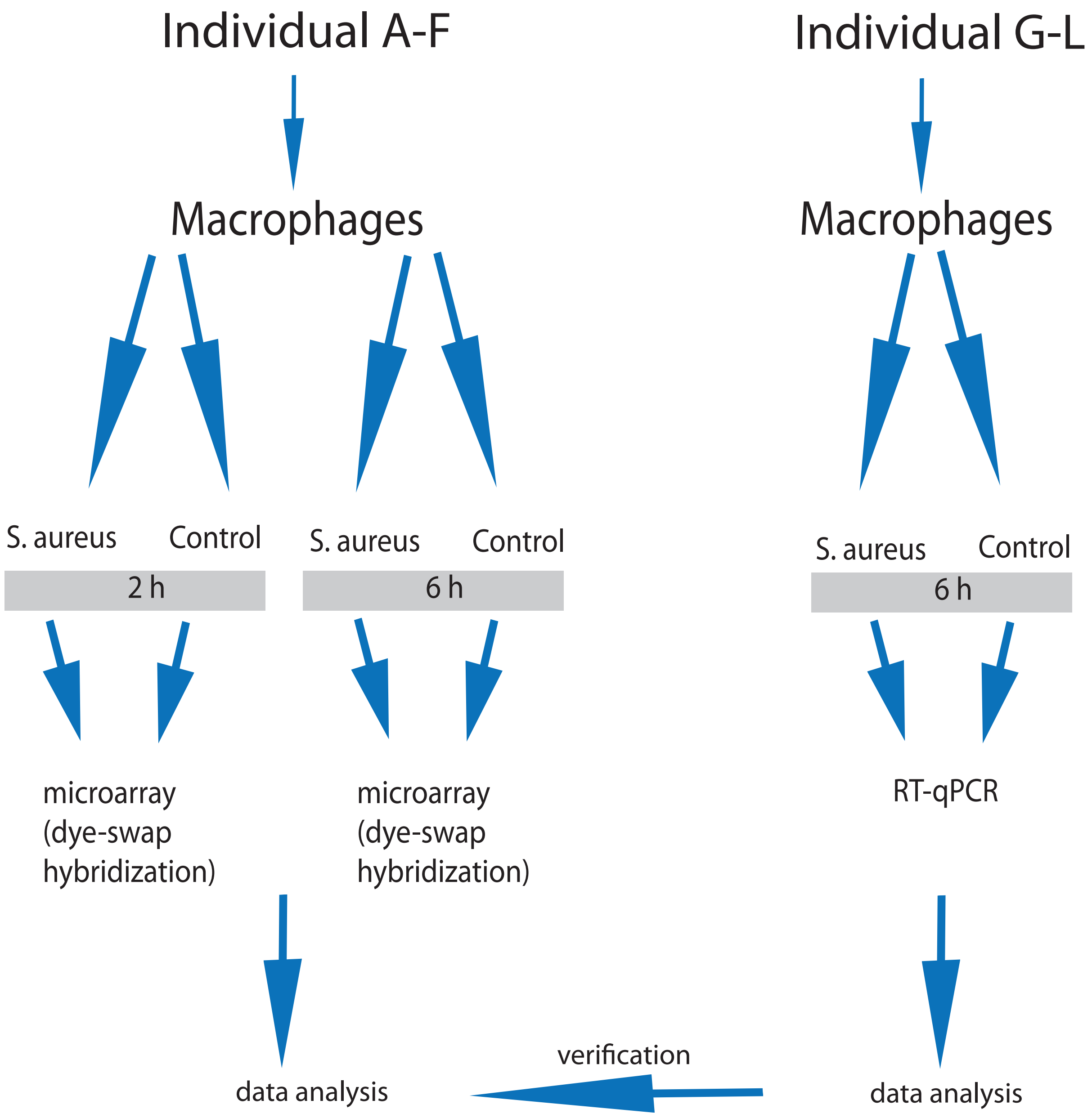

Individual A-F
Individual G-L
Macrophages
Macrophages
S. aureus
Control
2 h
S. aureus
Control
6 h
S. aureus
Control
6 h
microarray
(dye-swap
hybridization)
microarray
(dye-swap
hybridization)
RT-qPCR
data analysis
verification
data analysis
