# Supplementary material for: The early phase transcriptome of bovine monocyte-derived macrophages infected with Staphylococcus aureus in vitro
Source: BMC Genomics. 2013 Dec 17;14:891. doi: 10.1186/1471-2164-14-891 (PMC3878444; doi:10.1186/1471-2164-14-891)
Supplement: Additional file 7: Table S6 — List of primers used for reverse transcription-quantitative PCR (RT-qPCR). [file 1471-2164-14-891-S7.docx]

**Table S6**. List of primers used for reverse transcription-quantitative PCR (RT-qPCR).

| Gene symbol, accession no and name | Primers (5’🡪3’) | Amplicon (bp) |
| --- | --- | --- |
| ***BAD*** NM_001035459 BCL2-antagonist of cell death | CCCAGGTCTCAGCAAGCACT CGGTAGGAGCTGTGACGACT | 138 |
| ***CASP1*** BM288107 Caspase 1 | ACTGTTCCTTAGTGGCCCCAG AGCCGAATATGGGATGTCTCC | 129 |
| ***CCL5*** NM_175827 C-C motif chemokine 5 precursor | CTCCATGGCAGCAGTTGTCTTT AGCGTTGATGTACTCTCGCACC | 91 |
| ***CCR5*** NM_001011672 C-C chemokine receptor type 5 | TCTCGTCATACATGCAGCCCAC AGCACCAGCCCCAAGATGACTA | 95 |
| ***FOS***  NM_182786 v-FOS FBJ murine osteosarcoma viral oncogene homolog | GCAGAGCTCAGAGCATTGGC CCGCTTGGAGGGTGTCAGT | 150 |
| ***ICAM1*** NM_174348 Intercellular adhesion molecule-1 | CTGTGTGTGCTGAGGCCCTA TGTGGACAGTAATGCCGGAG | 121 |
| ***IFNb*** * (NM_174350) Interferon beta | CCTGTGCCTGATTTCATCATGA GCAAGCTGTAGCTCCTGGAAAG | 97 |
| ***IRF1*** NM_177432 Interferon regulatory factor 1 | ATCGAACGGACTCTCACTCCAG GGCGACACCTGAAAGTTGTACA | 125 |
| ***MAPK14*** NM_001102174 Mitogen-activated protein kinase 14, p38 | AGCCTGTGGAAGAAGAGCTCC TGATTACGGCCAAGTTCATGAA | 131 |
| ***PAK1*** NM_001076898 p21/Cdc42/Rac1-activated kinase 1 | CGGGTGATACTCAGCCAGGA TCCTCCCAAAAAGATGCTTGA | 132 |
| ***RSF1***  XM_002699063 Remodeling and spacing factor 1 | TAATAGCACCTGGGCGTGG CATCTTTGTCACGGCCAATTG | 185 |
| ***TLR8***  NM_001033937 Toll-like receptor 8 | TGCCAAGCTCGCTAACAGAA GGCAAGGTACACAGGGAAATG | 150 |
| ***TNFa***  NM_173966 Tumor necrosis factor alpha | TCTTCTCAAGCCTCAAGTAACAAG CCATGAGGGCATTGGCATAC | 103 |

* Derived from Lee, S. R., G. T. Pharr, B. L. Boyd, and L. M Pinchuk. 2008. Bovine viral diarrhea viruses modulate toll-like receptors, cytokines and co-stimulatory molecules genes expression in bovine peripheral blood monocytes. Comp. Immunol. Microb. 31:403–418.
